# Thermo-Responsive Polyion Complex of Polysulfobetaine and a Cationic Surfactant in Water

**DOI:** 10.3390/polym14153171

**Published:** 2022-08-03

**Authors:** Thu Thao Pham, Shin-ichi Yusa

**Affiliations:** Department of Applied Chemistry, Graduate School of Engineering, University of Hyogo, 2167 Shosha, Himeji 671-2280, Japan; phamthuthao.hus@gmail.com

**Keywords:** polymer-surfactant complex, polysulfobetaine, UCST behavior, electrostatic interaction, cationic surfactant

## Abstract

Poly(4-((3-methacrylamidopropyl)dimethylammonium)butane-1-sulfonate) (PSBP) was prepared via controlled radical polymerization. PSBP showed upper critical solution temperature (UCST) behavior in aqueous solutions, which could be controlled by adjusting the polymer and NaCl concentrations. Owing to its pendant sulfonate anions, PSBP exhibited a negative zeta potential of −7.99 mV and formed a water-soluble ion complex with the cationic surfactant cetyltrimethylammonium bromide (CTAB) via attractive electrostatic interaction. A neutral PSBP/CTAB complex was formed under equimolar concentrations of the pendant sulfonate group in PSBP and the quaternary ammonium group in CTAB. Transmittance electron microscopic images revealed the spherical shape of the complex. The stoichiometrically neutral-charge PSBP/CTAB complex exhibited UCST behavior in aqueous solutions. Similar to PSBP, the phase transition temperature of the PSBP/CTAB complex could be tuned by modifying the polymer and NaCl concentrations. In 0.1 M aqueous solution, the PSBP/CTAB complex showed UCST behavior at a low complex concentration of 0.084 g/L, whereas PSBP did not exhibit UCST behavior at concentrations below 1.0 g/L. This observation suggests that the interaction between PSBP and CTAB in the complex was stronger than the interpolymer interaction of PSBP.

## 1. Introduction

Polymer/surfactant complex systems have been extensively explored for many years [1,2,3,4,5] owing to their applicability within diverse fields, such as wastewater treatment [6], coatings [7], cosmetics [8,9], detergents [10], and drug delivery systems [11]. In particular, great attention has been paid to the formation of polymer/surfactant complexes through electrostatic interactions. As a result of these investigations, characteristics of the complexes, such as size and shape, are known to be influenced by the structure and molecular weight of the polymer and the properties of the surfactant [12].

Polybetaines are zwitterionic polymers containing both cationic and anionic moieties in the same pendant group [13]. Polybetaines can be classified into polycarboxybetaines, polysulfobetaines, and polyphosphobetaines [14,15,16]. Some of them exhibit pH-responsive [17,18,19,20,21] and thermo-responsive behaviors [22,23,24,25,26,27] and biocompatibility [28,29,30]. Especially, polysulfobetaines show upper critical solution temperature (UCST) behavior in water [31,32,33,34,35,36,37,38]. UCST is the critical temperature above which the mixture solution is miscible, meaning that a single phase exists for all of the composition, and under critical temperatures, the solution becomes turbid because the solute cannot dissolve in solvent. Accordingly, these polymers cannot dissolve in water below the UCST, whereas they dissolve in water above the UCST. For instance, a series of thermo-responsive polysulfobetaines, i.e., poly(3-((3-methacrylamidopropyl)dimethylammonio)propane-1-sulfonate) (PSPP), poly(2-hydroxy-3-((3-methacrylamidopropyl)dimethylammonio)propane-1-sulfonate) (PSHPP), and poly(4-((3-methacrylamidopropyl)dimethylammonio)butane-1-sulfonate) (PSBP), were synthesized via reversible addition–fragmentation chain-transfer (RAFT) radical polymerization. These polysulfobetaines exhibit UCST behavior in water, with the phase transition temperature (*T*_p_) of PSBP being much higher than that of PSPP and PSHPP. This might be related to the different lengths of the pendant alkyl spacer in the betaine moiety. The thermo-responsive behavior of these polysulfobetaines is influenced by the degree of polymerization (DP), polymer concentration (*C*_p_), salt concentration, and deuterated compounds, such as D_2_O [37,39]. In the preparation of complexes with polyelectrolytes, cetyltrimethylammonium bromide (CTAB) is a widely used cationic surfactant [40,41,42,43,44]. Thus, Fundin et al. reported the formation (in a diluted solution) of a complex of the polyanion sodium poly(styrenesulfonate) (PSSNa) and the oppositely charged surfactant CTAB [40]. The salt concentration and the ratio of PSSNa and CTAB affected the size and shape of the obtained PSSNa/CTAB complex. In the absence of salt, with an increased molar ratio, from 0.6 to 1.2, for CTAB/PSSNa, the hydrodynamic radius (*R*_h_) value of the complex reduced from 31.3 to 22.8 nm, indicating that the PSSNa coil strongly contracted to interact with CTAB, producing the PSSNa/CTAB complex by adding CTAB. Additionally, the *R*_h_ value of the complex decreased when NaBr was added, from 0 to 100 mM, because NaBr screened the electrostatic interaction of PSSNa and CTAB in the complex. Chen et al. [41]. reported a polyion complex (PIC) micelle of CTAB and a graft copolymer, poly(ethylene glycol)-graft-poly(aspartic acid) (PEG-*g*-PAsp). In water, the quaternary ammonium cation of CTAB electrostatically interacted with the anionic carboxylate groups in PAsp to form a PIC inner core covered by noncharged hydrophilic PEG outer shells. The size of the complex markedly depended on the content of PEG in PEG-*g*-PAsp; particularly, larger complexes were formed for smaller PEG contents.

The thermo-responsive PICs were developed as a promising candidate for potential applications such as controlled drug release [45] or fluorescent imaging [46]. For instance, a PIC micelle of poly(*t*-butyl acrylate-*co*-acrylic acid)-*b*-poly(*N*-isopropylacrylamide) [P(*t*BA-*co*-AA)-*b*-PNIPAM] and the graft copolymer chitosan-*g*-poly(*N*-isopropylacrylamide) (CS-*g*-PNIPAM) displayed a multilayer core–shell–corona structure, with a thermo-responsive PNIPAM outer corona. The PIC micelle can encapsulate the anticancer DOX in the hydrophobic inner core (P*t*BA) by hydrophobic interaction. The PNIPAM had a lower critical solution temperature (LCST)-polymer; below the LCST, PNIPAM was hydrophilic and protected the PIC micelles. However, above the LCST, PNIPAM was blocked and became hydrophobic, with the polymer chains shrinking and collapsing onto the core of the micelles, leading to the release of drug [45]. Despite the obvious interest in polymeric materials that respond to an environmental temperature [47], polymer/surfactant complexes, showing thermo-responsive behavior, have been scarcely explored [48]. For instance, Kim et al. [49] reported the formation of a complex between the sulfobetaine surfactant lauramidopropyl hydroxysultane (LAPHS) and cationic poly((3-(methacryloylamino)propyl)trimethylammonium chloride) (PMAPTAC). The LAPHS surfactant has a quaternary amino cation and a pendant sulfonate anion in its structure; however, it exhibits a negative charge when dissolved in water. Large aggregates of LAPHS and PMAPTAC were formed in water via electrostatic interactions. The resulting LAPHS/PMAPTAC complex showed UCST behavior, stemming from the electrostatic interaction between the cationic PMAPTAC and the sulfonate anion in LAPHS, with this UCST behavior controlled by tuning the concentration of the complex and the DP of PMAPTAC. Therefore, the LAPHS/PMAPTAC complex showed promising potential as a thermo-responsive material.

**Scheme 1:**
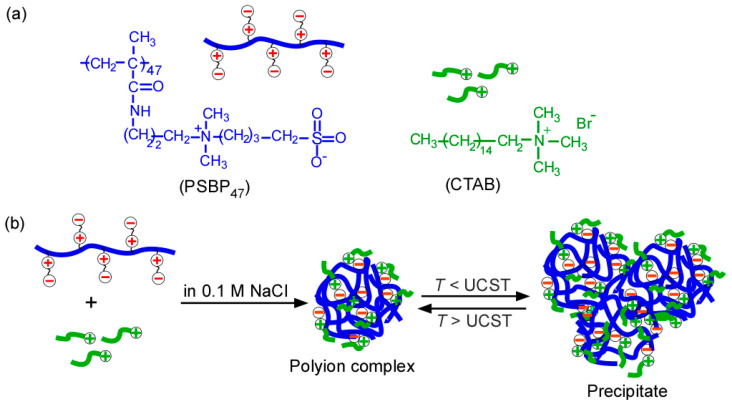
(**a**) Chemical structure of poly(4-((3-methacrylamidopropyl)dimethylammonio)butane-1-sulfonate) (PSBP) and cetyltrimethylammonium bromide (CTAB) and (**b**) the hypothetical structure and thermo-responsive behavior of the PSBP/CTAB polyion complex.

Herein, the formation of a complex between polysulfobetaine (PSBP) and CTAB as a cationic surfactant in an aqueous solution via electrostatic interactions is reported (Scheme 1). PSBP, with a DP of 47, was prepared via RAFT polymerization and was characterized via ^1^H nuclear magnetic resonance (NMR) spectroscopy and gel-permeation chromatography (GPC) measurements. PSBP, which contains pendant cationic quaternary ammonium and anionic sulfonate groups in its structure, shows biocompatibility, UCST behavior, and a zeta potential of −7.99 mV. Therefore, the formation of a complex of PSBP and CTAB in an aqueous solution via attractive electrostatic interaction was investigated. The resulting PSBP/CTAB complex exhibited thermo-responsive behavior comparable to that of PSBP. The formation and thermo-responsive behavior of the PSBP/CTAB complex in 0.1 M NaCl aqueous solution were studied using percentage transmittance (%*T*), dynamic light scattering (DLS), and transmittance electron microscope (TEM) techniques.

## 2. Materials and Methods

### 2.1. Material

4-((3-Methacrylamidopropyl)dimethylammonio)butane-1-sulfonate (SBP, 98%), azobis(4-cyanopentanoic acid) (V-501, 98%), and CTAB (98%) were purchased from Fujifilm Wako Pure Chemical (Osaka, Japan) and used without further purification. 4-Cyanopentanoic acid dithiobenzoate (CPD) was synthesized according to a previously reported method [50]. *N*-phenyl-1-naphthylamine (PNA, >98%) was purchased from Tokyo Chemical Industry (Tokyo, Japan) and used without further purification. Water was purified using an ion-exchange column system.

### 2.2. Preparation of PSBP

PSBP was prepared via RAFT polymerization as follows: SBP (5.85 g, 20.0 mmol), CPD (111.8 mg, 0.40 mmol) and V-501 (56.1 mg, 0.20 mmol) were dissolved in pure water (20 mL) at a [SBP]/[CPD]/[V-501] molar-feed ratio of 50/1/0.5. The polymerization was performed at 70 °C for 2 h under an argon atmosphere. The conversion was estimated to be 94.8%, according to the integral intensity ratio of the vinyl proton at 5.6 ppm and the pendant methylene protons at 2.9 ppm of SBP in the ^1^H NMR spectra, recorded before and after the polymerization. The solution obtained after the polymerization was dialyzed against pure water for one day and recovered by freeze-drying (4.96 g, 78.8%). The number-average molecular weight (*M*_n_(GPC)) and molecular weight distribution (*M*_w_/*M*_n_) of PSBP were determined to be 1.51 × 10^4^ g/mol and 1.09, respectively, via GPC measurement.

### 2.3. Preparation of the PSBP/CTAB Complex

PSBP at a *C*_p_ of 0.5 g/L (1.68 mM of SBP unit) and CTAB at 0.1 g/L (0.274 mM) were separately dissolved in 0.1 M NaCl aqueous solutions. The PSBP solution was added to the CTAB solution with constant stirring for 5 min. The molar ratio of the SBP units in PSBP and CTAB, in the mixed solution, was determined according to the positively charged CTAB mole fraction, i.e., mixing ratio (*f*^+^) = [CTAB]/([CTAB] + [SBP]), where [CTAB] and [SBP] are the molar concentrations of the CTAB and SBP units in the aqueous solution. The final concentrations of the SBP and CTAB units in the complex solution with an *f*^+^ of 0.5 were 0.0375 g/L (0.126 mM of the SBP unit) and 0.046 g/L (0.126 mM), respectively. The concentration of the complex (*C*_com_, g/L) was defined as the weigh concentration of the complex, estimated by the total weight of PSBP and CTAB divided by the volume of the complex solution, and was found to be 0.084 g/L. The PSBP/CTAB complex was prepared at a *C*_com_ of 0.084 g/L for further experiments unless otherwise noted.

### 2.4. Measurements

^1^H NMR measurements were performed in D_2_O using a JEOL (Tokyo, Japan) JNM-ECZ 400 MHz NMR instrument. The standard pulse program stebpgp1s19 was employed with a stimulated echo, bipolar gradient pulse, and a one spoil gradient with a 3-9-19 pulse sequence (WATERGATE) to suppress the water signal. The *M*_n_(GPC) and *M*_w_/*M*_n_ of PSBP were obtained via GPC measurements, using a mixture of 50 mM phosphate buffer at a pH of 9 and acetonitrile (9/1, *v*/*v*) as an eluent. The GPC signal was detected using a Tosoh (Tokyo, Japan) RI-8020 refractive index detector, working at 40 °C with a Tosoh (Tokyo, Japan) DP-8020 pump and a Shodex (Tokyo, Japan) GF-7M column. To estimate the *M*_n_ and *M*_w_/*M*_n_ values, a calibration curve, prepared using standard PNaSS samples, was applied. The hydrodynamic radius (*R*_h_), light scattering intensity (LSI), and zeta potential of the samples were obtained using a Malvern (Kobe, Japan) Nano ZS with a He–Ne laser (4 mW at 632.8 nm). Before the measurements, the sample solutions were filtered through a poly(tetrafluoroethylene) membrane filter with a 0.2 μm pore size. The formation of the PIC aggregates was confirmed via transmission electron microscopy (TEM) observation, using a JEOL (Tokyo, Japan) JEM-2100 microscope. A PSBP/CTAB complex with an *f*^+^ of 0.5 in 0.1 M NaCl was prepared. Then, a drop of the sample solution was put onto a copper grid coated with Formvar thin films. Excess complex solution was blotted using filter paper. Subsequently, the sample was stained with sodium phosphotungstate and dried under vacuum overnight. UCST behavior was monitored using a Jasco (Tokyo, Japan) V-730 UV–vis spectrophotometer, at 700 nm, with a Jasco ETC-7171 temperature controller at a heating/cooling rate of 1.0 °C/min.

## 3. Results

### 3.1. Preparation of PSPB

PSBP was prepared via RAFT polymerization with a conversion (*p*) of 94.8%. The theoretical DP (DP(theo)) and theoretical *M*_n_ (*M*_n_(theo)) were estimated using the following formulas:

(1) $$\mathrm{DP}\left(\mathrm{theo}\right)=\frac{{\left[\mathrm{SBP}\right]}_{0}}{{\left[\mathrm{CPD}\right]}_{0}}\times \;p\;$$

(2) $$M_{n}{(\mathrm{theo})=\mathrm{DP}}_{\mathrm{theo}}{\times M}_{\mathrm{SBP}}{+M}_{\mathrm{CPD}}$$

where [SBP]_0_ and [CPD]_0_ are the initial SBP and CPD concentrations, respectively, and *M*_SBP_ and *M*_CPD_ are the molecular weights of SBP and CPD, respectively. The DP(theo) and *M*_n_(theo) were calculated to be 47 and 1.40 × 10^4^ g/mol, respectively. The DP of PSBP could not be determined using ^1^H NMR because the proton signal of the pendant amide group overlapped with that of the terminal phenyl proton derived from CPD at 7.8 ppm in D_2_O (Supplementary Materials Figure S1). The GPC elution curve for PSBP was unimodal (Supplementary Materials Figure S2), with an *M*_n_(GPC) of 1.51 × 10^4^ g/mol and an *M*_w_/*M*_n_ of 1.09. These results suggest that the obtained polymer exhibited a defined controlled structure [51,52,53]. The calculated DP(theo), *M*_n_(theo), and *M*_w_/*M*_n_ are listed in Table 1.

### 3.2. Preparation and Characterization of the Polymer/Surfactant Complex

**Figure 1:**
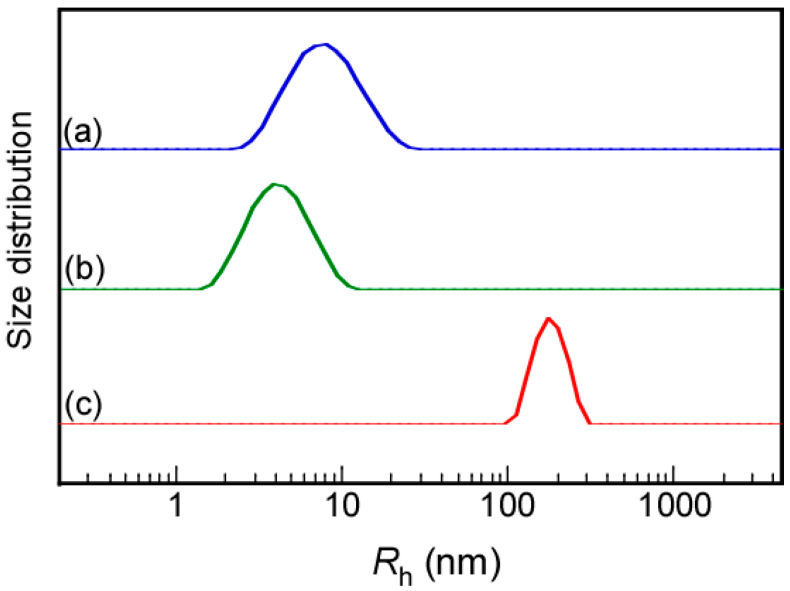
Hydrodynamic radius (*R*_h_) distributions of (**a**) PSBP at 2.0 g/L (6.72 mM of SBP unit), (**b**) CTAB at 10 g/L (27.4 mM), and (**c**) the PSBP/CTAB complex, with a mixing ratio of 0.5 within a complex concentration of 0.084 g/L in a 0.1 M NaCl aqueous solution at 25 °C.

The zeta potential of PSBP was −7.99 mV. Some sulfobetaine polymers have been found to exhibit a negative charge despite containing both cationic and anionic charges in their structure. For instance, a poly(*N*-(3-acrylamidopropyl)-*N*,*N*-dimethylammonio propylsulfonate)-*ran*-ω-perfluoroalkyl poly(ethylene glylcol) acrylate)-block-polystyrene ((PAAmPrDMAPS-*r*-R_f_PEGA)-*b*-PS) block copolymer showed a zeta potential ranging from −10 to −40 mV, within a pH range of 1–10 [54]. The negative charge was attributed to PAAmPrDMAPS because the R_f_PEGA and PS units have no charged groups. PSBP interacted with cationic CTAB, with a zeta potential of 8.62 mV via electrostatic interactions, to form a PIC. The *R*_h_ distributions of the PSBP, CTAB, and PSBP/CTAB complex were unimodal (Figure 1). The *R*_h_ value of PSBP was 6.4 nm, suggesting that it dissolved as a unimer in water. The DLS measurement of CTAB was performed at a concentration of 10 g/L (27.4 mM), which is above its critical micelle concentration (cmc) of 0.9 mM [55,56]. Hence, an *R*_h_ value of 3.6 nm obtained for CTAB indicated the size of the micelle. The LSI values for PSBP, at a *C*_p_ of 2.0 g/L (6.72 mM of SBP unit), and CTAB, at 10 g/L (27.4 mM), were 135.5 and 106.0 kcps, respectively. After mixing, the final concentration of PSBP in the complex solution was 0.038 g/L (0.126 mM of SBP unit) and 0.046 g/L (0.126 mM) for CTAB. The mixture of PSBP and CTAB in a 0.1 M NaCl aqueous solution afforded an *R*_h_ value of 165.5 nm, suggesting the formation of a PIC. The LSI value of the PSBP/CTAB complex was 354.5 kcps, which was larger than that of PSBP and CTAB; however, it was not sufficiently large to fit with the large size of the complex. This might have been caused by the small concentration of PSBP and CTAB in the complex at a *C*_com_ of 0.084 g/L. The zeta potential of the PSBP/CTAB mixture was close to zero (1.46 × 10^−3^ mV), demonstrating that PSBP and CTAB interacted electrostatically to form a stoichiometrically neutral PIC.

**Figure 2:**
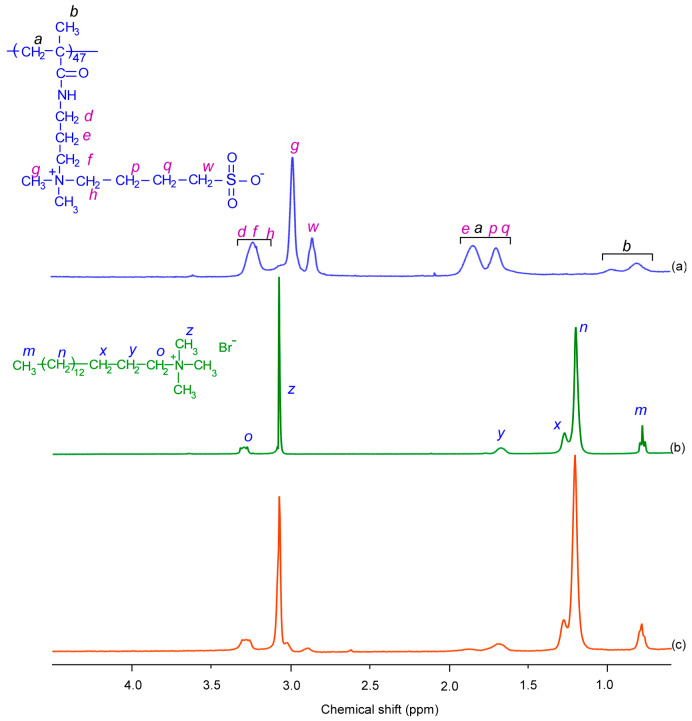
^1^H NMR spectra of (**a**) PSBP at 2.0 g/L (6.72 mM of SBP), (**b**) CTAB at 2.0 g/L (5.48 mM), and (**c**) the PSBP/CTAB complex with a mixing ratio of 0.5 at 25 °C within a complex concentration of 0.69 g/L in D_2_O containing 0.1 M NaCl.

Figure 2 shows the ^1^H NMR spectra of PSBP, CTAB, and the PSBP/CTAB complex in D_2_O. For the spectrum of PSBP (Figure 2a), the resonance band at 0.81–0.97 ppm (peak *b*) can be attributed to the main chain protons, and the signal at 2.85 pm (peak *w*) corresponds to the pendant methylene protons near the sulfonate group. Meanwhile, the spectrum of CTAB showed signals attributable to the methyl protons in the quaternary amino group for 3.02 ppm (peak *z*) and methylene protons in the alkyl chain at 1.16, 1.21, and 1.62 ppm (Figure 2b). For the ^1^H NMR spectrum of the mixture of PSBP and CTAB in D_2_O, all the PSBP signals in the complex were clearly broad, demonstrating that the mobility of PSBP was restricted. The signals for CTAB were slightly broadened. The ratios of the full width at haff maxima (FWHM) of peaks *n* (1.16 ppm) and *z* (3.02 ppm) for CTAB, before and after mixing, were 2.4 and 3.3, respectively. The larger FWHM observed after mixing suggests that the anionic group from PSBP interacted with the cationic CTAB molecules.

**Figure 3:**
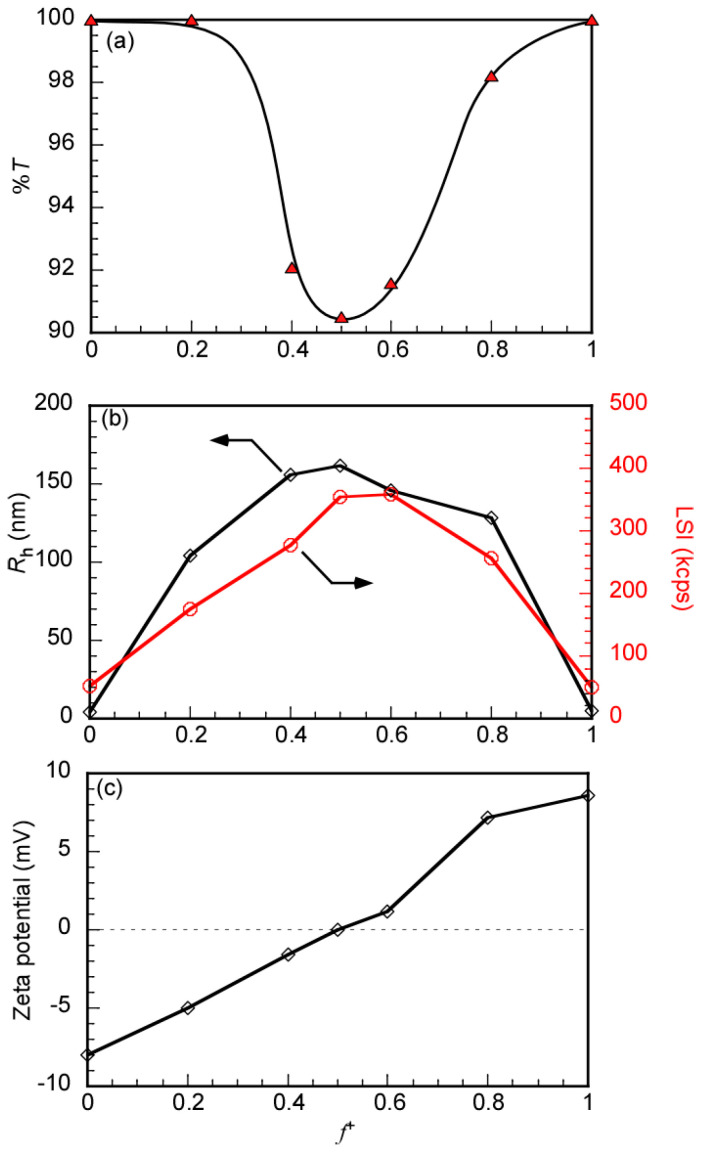
(**a**) Percentage transmittance (%*T*), (**b**) hydrodynamic radius (*R*_h_, black diamonds), light scattering intensity (LSI, red circles), and (**c**) the zeta potential of 0.1 M NaCl aqueous PSBP/CTAB complex solutions as a function of the mixing ratio (*f*^+^) at 25 °C.

The %*T*, *R*_h_, LSI, and zeta potential of the PSBP/CTAB complex were measured as a function of *f*^+^ to understand the effect of the polymer/surfactant ratio of the complex (Figure 3). The solubility of the complex in a 0.1 M NaCl aqueous solution can be expressed in terms of the %*T* value. A minimum %*T* value of 90.5% was obtained at an *f*^+^ of 0.5, suggesting the formation of the largest PSBP/CTAB complex. Additionally, at an *f*^+^ of 0.5, the *R*_h_ and LSI reached the maximum values of 165.5 nm and 354.5 kcps, respectively, which are in agreement with the %T. Furthermore, a close to zero zeta potential of 1.46 × 10^−3^ mV was obtained at an *f*^+^ of 0.5, indicating the neutralization of the anionic group in PSBP and the cationic group in CTAB. These results indicate that the largest complex was obtained when the charges of PSBP and CTAB were balanced. In addition, the *R*_h_ values for the complex, at an *f*^+^ near to 0.5, were close. The *R*_h_ was calculated at 155 and 145 nm for *f*^+^ = 0.4 and 0.6, respectively. However, the size of the complex at *f*^+^ = 0.2 (*R*_h_ = 104.4 nm) was smaller than that at *f*^+^ = 0.8 (*R*_h_ = 128.5 nm); this might be explained by an excess of CTAB which still existed in the complex solution. The hydrophobic tail of CTAB may attach to hydrophobic methylene groups from the PSBP side chains, leading to the formation of a larger complex. The zeta potential of the PSBP/CTAB complex increased with increasing *f*^+^, and was the result of free cationic surfactant in the solution.

**Figure 4:**
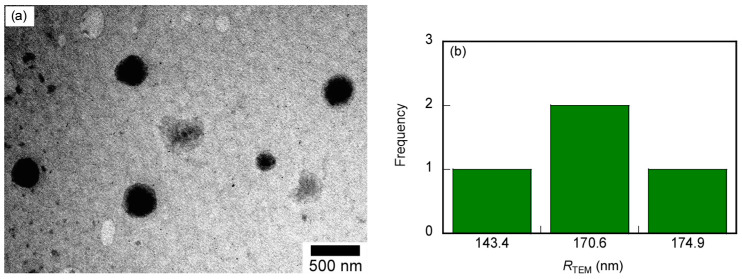
(**a**) TEM image and (**b**) histogram of particle size distribution of PSBP/CTAB complex with a mixing ratio of 0.5 within a complex concentration of 0.084 g/L in a 0.1 M NaCl aqueous solution.

The shape and size of the PSBP/CTAB complex were studied via TEM observations (Figure 4). The resulting average radius of the complex was 164.9 nm, which was close to the 165.5 nm *R*_h_ value, estimated via DLS measurements.

To gain more insight into the structure of the PSBP/CTAB complex, fluorescence measurements were performed using PNA as a hydrophobic fluorescence probe. The maximum fluorescence wavelength (*λ*_max_) and fluorescence intensity (FI) of PNA are influenced by the microenvironmental polarity around the PNA molecules. The *λ*_max_ shifts to a shorter wavelength and the FI of PNA increases in a hydrophobic environment [57]. The PNA fluorescence was recorded in the absence and presence of PSBP, CTAB, and the PSBP/CTAB complex, within 0.1 M aqueous solutions (Supplementary Materials Figure S3). The *λ*_max_ and FI values of PNA in a pure 0.1 M aqueous solution were 463.8 nm and 6.8, respectively. The *λ*_max_ values of PSBP, CTAB, and the PSBP/CTAB complex were 460.2, 450.6, and 451.2 nm, respectively, and the FI values in the presence of PSBP, CTAB, and the PSBP/CTAB complex were 9.9, 26.0, and 11.2, respectively. These results suggest that PSBP has almost no hydrophobic part that can encapsulate the PNA molecules. At a concentration of 0.05 g/L, which is below its cmc value of 0.328 g/L [55], CTAB could not form micelles. PNA may interact with the hydrophobic alkyl chain in CTAB.

PSBP is a sulfobetaine polymer, showing UCST behavior in water (Supplementary Materials Figure S4), which can be controlled by adjusting *C*_p_ and NaCl concentrations ([NaCl]). The transition temperature (*T*_p_) of PSBP decreased from 35.5 °C to 6.9 °C with a decreasing *C*_p_ of 5.0 g/L (16.8 mM of SBP unit) to 1.0 g/L (3.36 mM of SBP unit) (Figure S5). At a *C*_p_ equal to or below 0.5 g/L, PSBP did not show thermo-responsive behavior. Besides, the *T*_p_ of PSBP at a *C*_p_ of 5.0 g/L shifted from 35.5 °C to 4 °C upon increasing additions of [NaCl] from 0 to 0.05 M (Supplementary Materials Figure S6). At a [NaCl] concentration of above 0.07 M, PSBP did not exhibit thermo-responsive behavior. To investigate the thermo-responsive behavior of the PSBP/CTAB complex, the %*T* of a 0.1 M NaCl aqueous complex solution with an *f*^+^ of 0.5 was measured as a function of the temperature upon heating and cooling (Supplementary Materials Figure S7). The complex showed UCST behavior at a *T*_p_ of 5.9 °C and 20 °C during heating and cooling cycles, respectively. However, unlike the heating process, the %*T* and temperature plot obtained in the cooling process always overlapped by multiple cycles. Therefore, to study the UCST behavior of the complex system, the cooling process was investigated in detail.

**Figure 5:**
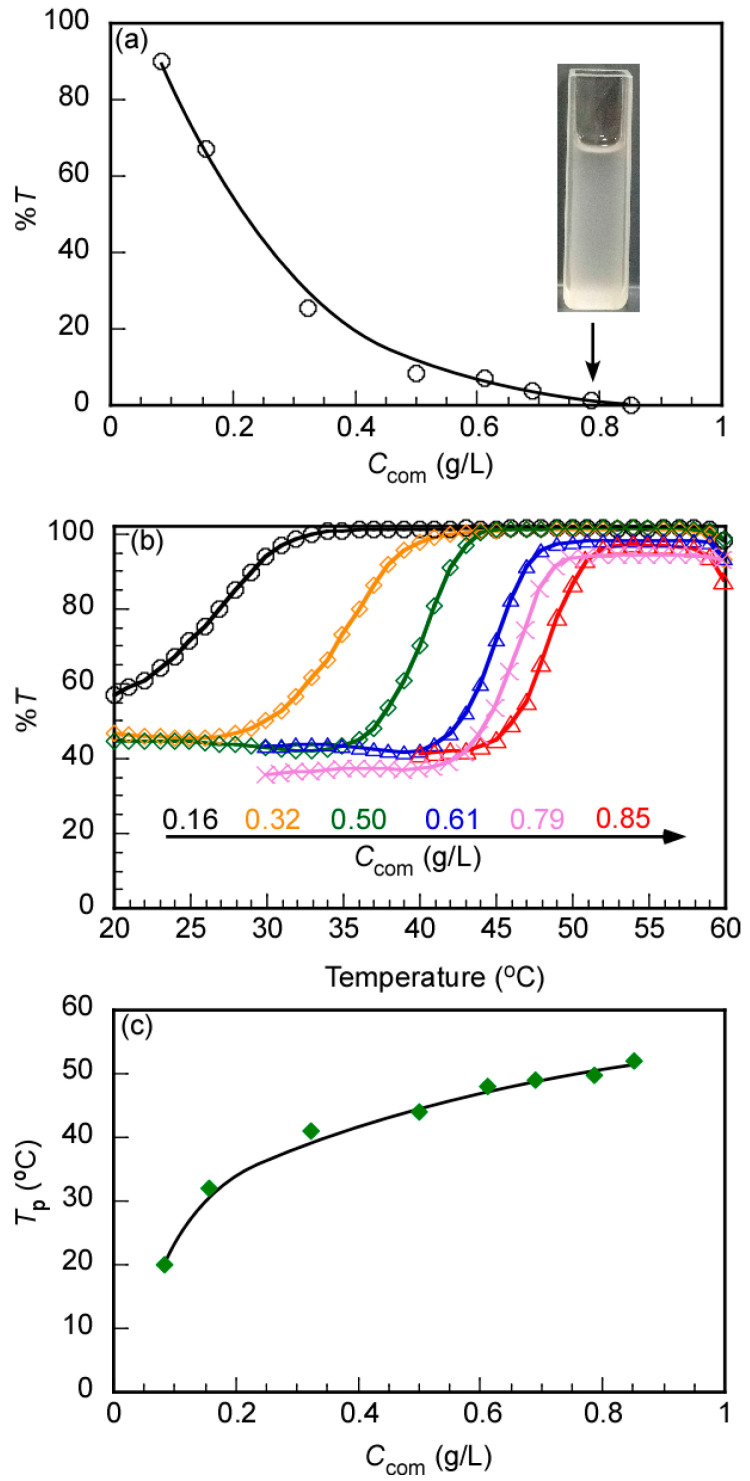
Percentage transmittance (%*T*) of the 0.1 M NaCl aqueous PSBP/CTAB complex solutions at 25 °C with a mixing ratio of 0.5 as a function of (**a**) the complex concentration (*C*_com_) and (**b**) the temperature at different *C*_com_ values of 0.16, 0.32, 0.50, 0.61, 0.79, and 0.85 g/L, and (**c**) the phase transition temperature (*T*_p_) dependant on *C*_com_ for the PSBP/CTAB complex.

The effect of *C*_com_ on the UCST behavior of the PSBP/CTAB complex was studied by plotting the %*T* of the PSBP/CTAB complex (in 0.1 M NaCl aqueous solution) as a function of *C*_com_ (Figure 5a) while keeping the *f*^+^ value constant at 0.5. With an increasing *C*_com_, from 0.084 to 0.85 g/L, the %*T* decreased from 90.5% to 0%, suggesting the formation of larger aggregates. Unfortunately, the *R*_h_ and LSI values of the complex could not be obtained because the complex solutions were too turbid under concentrations above 0.085 g/L. The %*T* of the 0.1 M NaCl aqueous complex solutions was also measured as a function of temperature at varying *C*_com_ (Figure 5b). Plotting *T*_p_ against *C*_com_ (Figure 5c) showed a rise in *T*_p_ from 20 °C to 51.9 °C with an increasing *C*_com_ (from 0.084 to 0.85 g/L). When C_com_ increased, the polymer and surfactant formed large aggregates. Therefore, breaking the electrostatic interactions required much more energy, causing a remarkable growth in the *T*_p_ value. This increase in *T*_p_ at high *C*_com_ was similar to that of PSBP without CTAB. As discussed above, the *T*_p_ value of PSBP also increased with increasing *C*_p_. However, the *T*_p_ value of the PSBP/CTAB complex at a *C*_com_ of 0.85 g/L was 51.9 °C. Meanwhile, for a *C*_p_ below 0.5 g/L, the PSBP did not exhibit UCST behavior (Supplementary Materials Figure S5). This indicates that the electrostatic interaction between PSBP and CTAB was stronger than the intra- and interpolymer chain interactions of PSBP.

**Figure 6:**
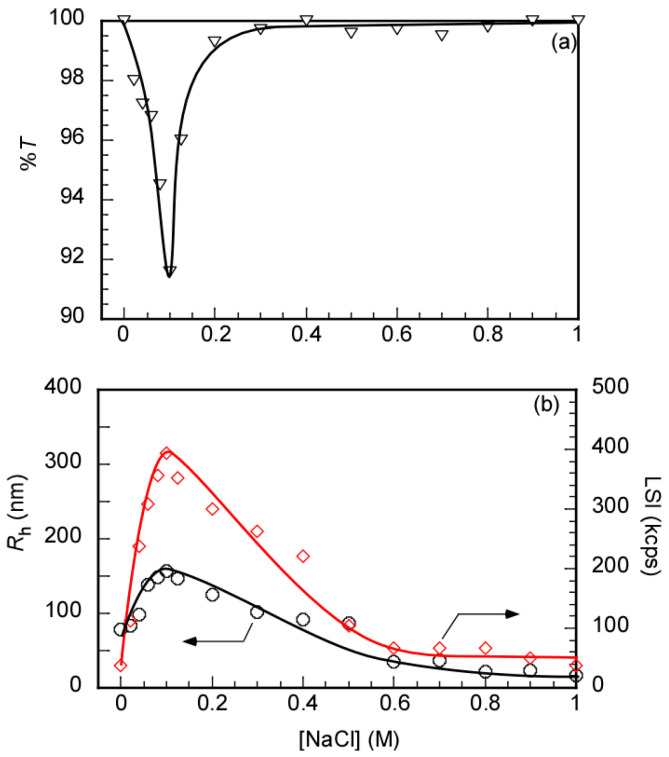
(**a**) Percentage transmittance (%*T*), (**b**) hydrodynamic radius (*R*_h_, black circles), and light scattering intensity (LSI, red diamonds) of aqueous PSBP/CTAB complex solutions as a function of [NaCl] at 25 °C.

**Figure 7:**
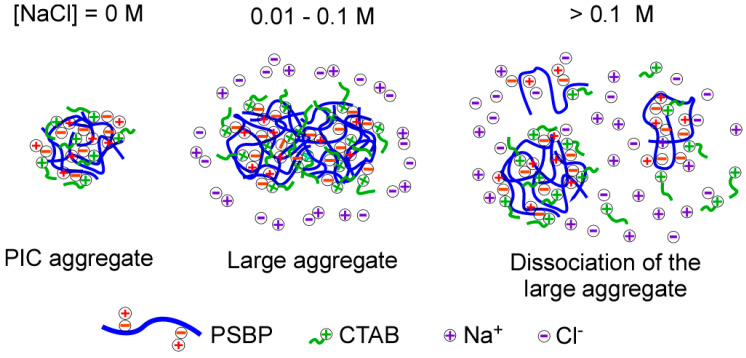
Schematic illustration of the effect of [NaCl] on the formation of the PSBP/CTAB complex.

The addition of NaCl affected the formation of the PSBP/CTAB complex due to the electrostatic screening effect of NaCl. To study the effect of [NaCl], a PSBP/CTAB complex, with an *f*^+^ of 0.5 and a *C*_com_ of 0.084 g/L, was prepared in pure water, and a predetermined amount of NaCl was then added to obtain NaCl-containing aqueous complex solutions. The [NaCl] dependence of %*T*, *R*_h_, and LSI was calculated (Figure 6). In the absence of NaCl, the solution was transparent, and the %*T*, *R*_h_, and LSI values were 100%, 78.2 nm, and 37.8 kcps, respectively, suggesting the formation of an PIC. At 0 M < [NaCl] ≤ 0.1 M, the solubility of the complex decreased because the %T decreased from 100 to 91.6% (Figure 6a). Moreover, the *R*_h_ and LSI increased to 156.7 nm and 393.8 kcps, respectively, for 0.1 M NaCl, implying the formation of large aggregates. In this [NaCl] range, the PSBP/CTAB complex may be influenced by the salting-out effect, leading to the liquid–liquid phase separation phenomena and the formation of large aggregates [58,59]. At 0.1 M < [NaCl] < 0.6 M, the %T value of the complex increased with increasing [NaCl] until reaching nearly 100%, whereas the *R*_h_ and LSI values decreased significantly. Upon the further addition of NaCl, Na^+^ and Cl^−^ replaced the cationic surfactant and the anionic groups of PSBP, leading to the dissociation of the large aggregates into smaller PIC micelles. Therefore, the dissociation of the PSBP/CTAB complex upon the addition of NaCl was evaluated. The %*T* of the PSBP/CTAB complex with an *f*^+^ of 0.5 at 0.1 M NaCl was found to be 91.6% (Figure 6a), which was slightly larger than that observed in Figure 3a (%*T* = 90.5%) because of the difference in the preparation of the complex. To study the effect of [NaCl] on the formation of the complex, a complex solution was prepared in pure water, with NaCl added up to an [NaCl] of 0.1 M. Moreover, to investigate the influence of the mixing ratio (Figure 3), complex solutions were prepared in 0.1 M NaCl. Using these two distinct methods for the preparation of the complex led to slightly different %*T*, *R*_h_, and LSI values. At an [NaCl] of 1.0 M, the *R*_h_ value decreased to 16.2 nm, suggesting that the complex was completely dissociated (Figure 6b). A similar trend was observed for the LSI value. This indicates that the electrostatic interaction of PSBP and CTAB was affected by [NaCl]. The effect of NaCl on the formation of the PSBP/CTAB complex is illustrated in Figure 7.

**Figure 8:**
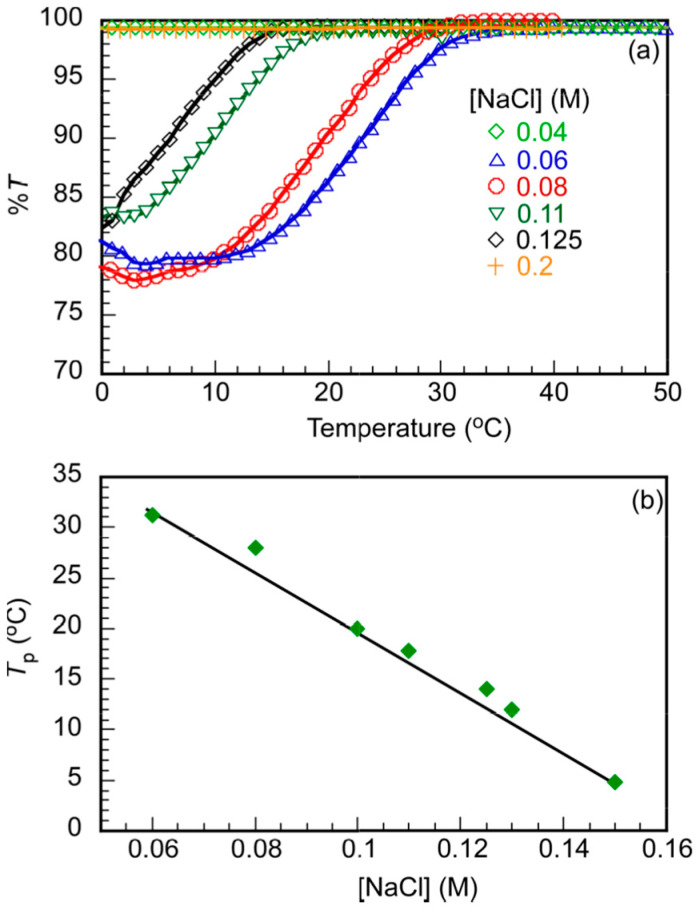
(**a**) Percentage transmittance (%*T*) of PSBP/CTAB complex aqueous solutions at a complex concentration of 0.084 g/L with a mixing ratio of 0.5 at different [NaCl], as a function of temperature after the cooling process and (**b**) [NaCl] dependence of the phase transition temperature (*T*_p_) for the PSBP/CTAB complex.

The UCST behavior of sulfobetaine polymers is known to be dependent on the salt concentration. In the present study, the *T*_p_ of PSBP at a *C*_p_ of 5.0 g/L was plotted against [NaCl], ranging from 0 to 0.05 M (Supplementary Materials Figure S6). The *T*_p_ value of PSBP was 35.5 °C without NaCl, and a drastic decrease to 4 °C was observed for [NaCl] 0.05 M. Above this concentration, PSBP did not show thermo-responsive behavior. This large effect of a small amount of NaCl on the *T*_p_ value was previously observed for PSBP and PSHPP [37]. Likewise, the UCST behavior of the PSBP/CTAB complex depended on [NaCl]. The %*T* of the complex solution at a *C*_com_ of 0.084 g/L was measured against the temperature after a cooling process at different [NaCl] (Figure 8). However, different from PSBP, the PSBP/CTAB complex did not show UCST behavior at [NaCl] below 0.06 M and equal to or above 0.2 M. Therefore, the *T*_p_ value of the PSBP/CTAB complex was studied in the range of 0.06 M ≤ [NaCl] ≤ 0.15 M (Figure 8b). This indicated that [NaCl] strongly influenced the *T*_p_ value of the PSBP/CTAB complex. At an [NaCl] of 0.06 M, the *T*_p_ of the PSBP/CTAB complex was 31.2 °C, whereas it decreased to 4.8 °C for an [NaCl] of 0.15 M. This might be due to the counterions, Na^+^ and Cl^−^, replacing the anionic and cationic groups in PSBP and CTAB [60], which would partially reduce the total energy required to break the electrostatic interactions that form the complex.

## 4. Conclusions

PSBP, with a well-defined structure, was prepared via RAFT polymerization. The negative zeta potential of PSBP suggested that it could form PIC aggregates with a cationic surfactant (CTAB) via electrostatic interactions. The charges of PSBP and CTAB were neutralized at an *f*^+^ of 0.5, affording the maximum values of Rh and LSI and a zeta potential of 0 mV. TEM observations revealed the spherical shape of the PSBP/CTAB complex. In aqueous solutions, the PSBP/CTAB complex exhibited UCST behavior, which was significantly affected by the complex concentration and [NaCl] in a similar manner to the UCST behavior of PSBP. However, at a low *C*_com_ of 0.084 g/L, the PSBP/CTAB complex showed UCST behavior, whereas PSBP did not exhibit thermo-responsive behavior below 1.0 g/L. These observations suggest that the interaction between PSBP and CTAB in the complex was stronger than the inter- and intra-polymer interactions of PSBP.

## Figures and Tables

**Table 1:** Degree of polymerization (DP), number-average molecular weight (*M*_n_), and molecular weight distribution (*M*_w_/*M*_n_).

Sample	DP	*M*_n_(theo) ^a^	*M*_n_(GPC) ^b^	*M*_w_/*M*_n_
	(theo)	× 10^−4^ (g/mol)	× 10^−4^ (g/mol)	
PSPB_47_	47	1.40	1.51	1.09

^a^ Calculated using Equation (2); ^b^ Obtained from GPC.

## Data Availability

The data used to support the findings of this study are available from the corresponding authors upon request.

## Supplementary Materials

The following supporting information can be downloaded at: https://www.mdpi.com/article/10.3390/polym14153171/s1, Figure S1. ^1^H NMR spectrum of PSBP in D_2_O at 25 °C; Figure S2. Gel-permeation chromatography (GPC) elution curve of PSBP obtained using a refractive index (RI) detector working at 40 °C and phosphate buffer as an eluent; Figure S3. Fluorescence spectra of PNA only (---) and PNA in the presence of PSBP at a concentration of 0.5 g/L (—), CTAB at a concentration of 0.05 g/L (—), and PSBP/CTAB at a concentration of 0.084 g/L (—) in 0.1 M aqueous solutions; Figure S4. Percent transmittance (%*T*) of an aqueous PSBP at a concentration of 3.0 g/L as a function of temperature upon heating and cooling processes; Figure S5. (a) Percent transmittance (%*T*) of aqueous PSBP solutions as a function of temperature at different polymer concentrations (*C*_p_) and (b) *C*_p_ dependence of the phase transition temperature (*T*_p_) of an aqueous PSPB solution; Figure S6. (a) Percent transmittance (%*T*) of aqueous PSBP solutions as a function of temperature at different NaCl concentration ([NaCl]) and (b) [NaCl] dependence of the phase transition temperature (*T*_p_) of an aqueous PSPB solution at a concentration of 5.0 g/L; Figure S7. Percent transmittance (%*T*) of a 0.1 M NaCl aqueous PSBP/CTAB complex solution with a mixing ratio of 0.5 as a function of temperature upon heating and cooling processes at a complex concentration of 0.084 g/L.
