# Supplementary material for: Thermo-Responsive Polyion Complex of Polysulfobetaine and a Cationic Surfactant in Water
Source: Polymers (Basel). 2022 Aug 3;14(15):3171. doi: 10.3390/polym14153171 (PMC9370920; doi:10.3390/polym14153171)
Supplement: Supplementary file 1 [file polymers-14-03171-s001.zip › polymers-1833338-supplementary.pdf]

Supporting information

Thermo-responsive polyion complex of polysulfobetaine and a cationic surfactant in water

Thu Thao Pham and Shin-ichi Yusa*

Department of Applied Chemistry, Graduate School of Engineering, University of Hyogo, 2167 Shosha, Himeji, Hyogo 671-2280, Japan

*Corresponding author

Shin-ichi Yusa

yusa@eng.u-hyogo.ac.jp

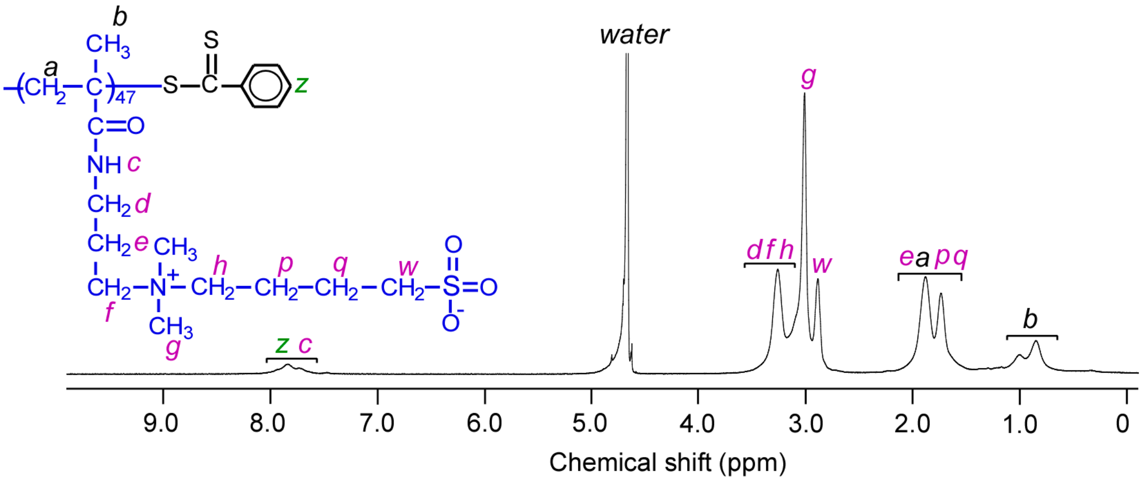

Figure S1. ^{1}H NMR spectrum of PSBP in D_2O at 25°C.

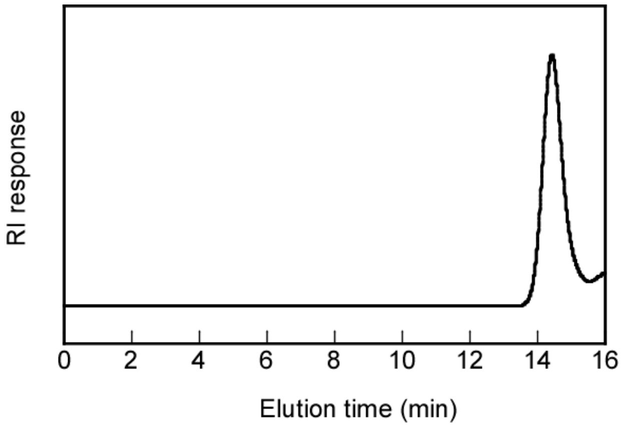

Figure S2. Gel-permeation chromatography (GPC) elution curve of PSBP obtained using a refractive index (RI) detector working at 40°C and phosphate buffer as an eluent.

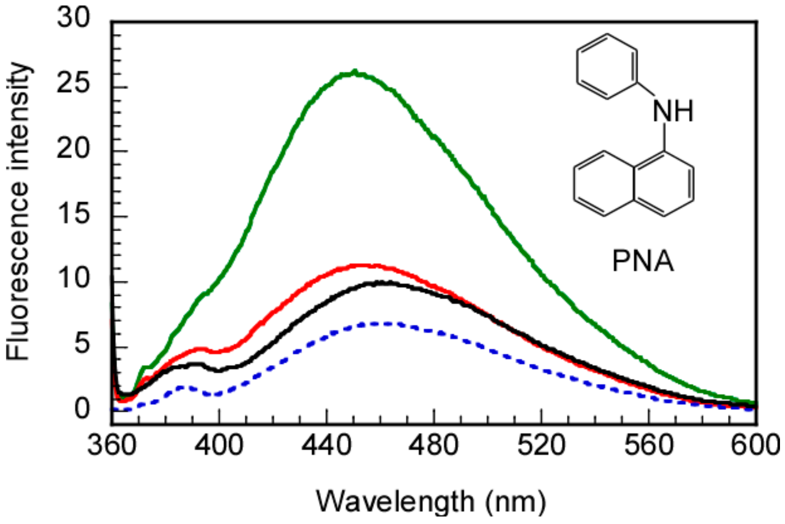

Figure S3. Fluorescence spectra of PNA only (---) and PNA in the presence of PSBP at a concentration of 0.5 g/L (—), CTAB at a concentration of 0.05 g/L (—), and PSBP/CTAB at a concentration of 0.084 g/L (—) in 0.1 M aqueous solutions.

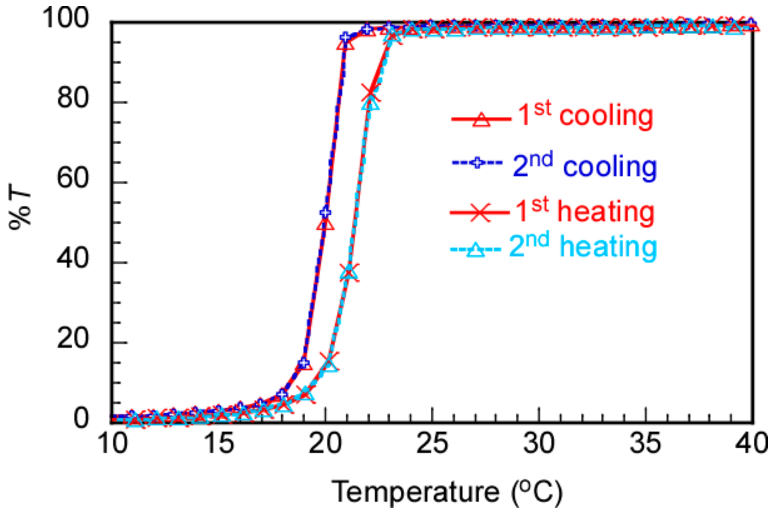

Figure S4. Percent transmittance (%T) of an aqueous PSBP at a concentration of 3.0 g/L as a function of temperature upon heating and cooling processes.

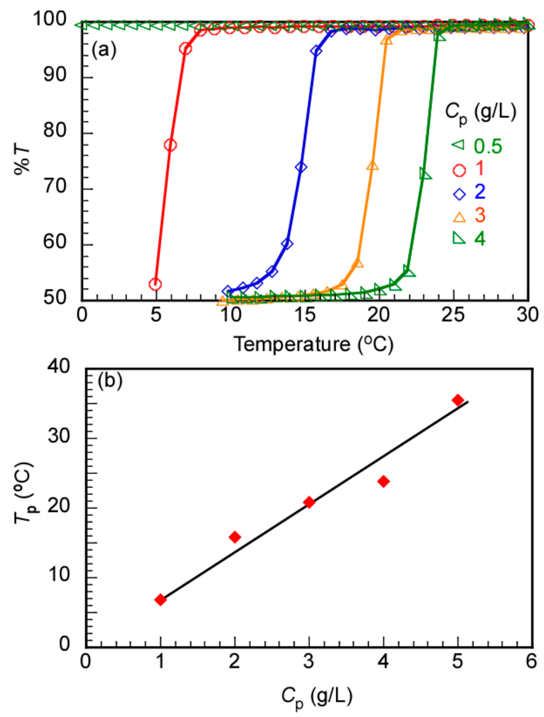

Figure S5. (a) Percent transmittance (%*T*) of aqueous PSBP solutions as a function of temperature at different polymer concentrations (C_p) and (b) C_p dependence of the phase transition temperature (T_p) of an aqueous PSPB solution.

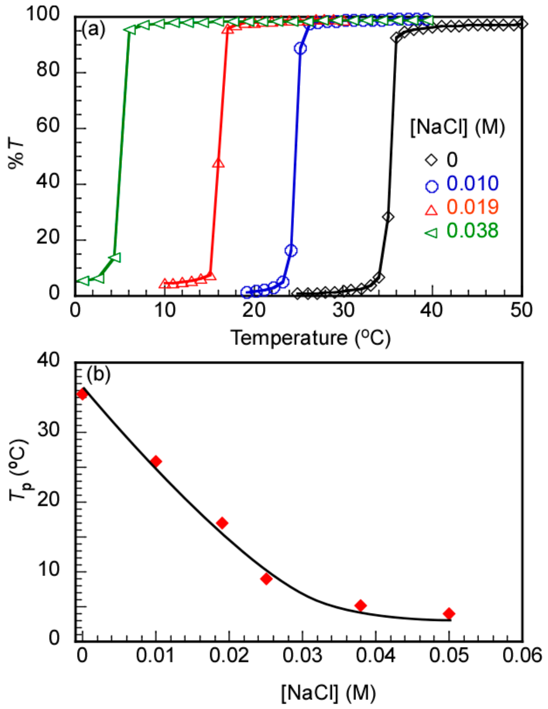

Figure S6. (a) Percent transmittance (%*T*) of aqueous PSBP solutions as a function of temperature at different NaCl concentration ([NaCl]) and (b) [NaCl] dependence of the phase transition temperature (T_P) of an aqueous PSPB solution at a concentration of 5.0 g/L.

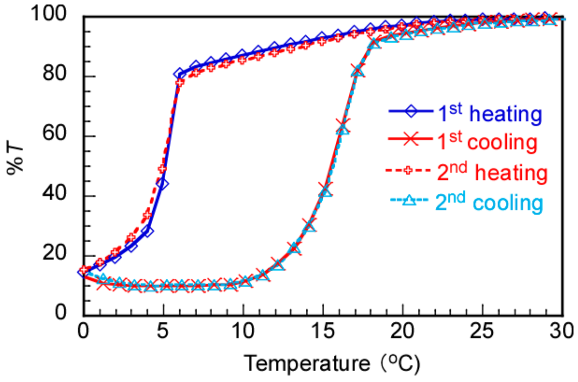

Figure S7. Percent transmittance (%*T*) of a 0.1 M NaCl aqueous PSBP/CTAB complex solution with a mixing ratio of 0.5 as a function of temperature upon heating and cooling processes at a complex concentration of 0.084 g/L.
